# Research Progress of Combining High Tibial Osteotomy with Platelet-rich Plasma for Osteoarthritis Therapy

**DOI:** 10.7150/ijms.114227

**Published:** 2025-07-10

**Authors:** Xiangdong Wen, Yichen Zhang, Xingchen Wei, Yang Su, Jinquan Cui, Senbo An, Shui Sun

**Affiliations:** 1 Department of Joint Surgery, Shandong Provincial Hospital, Shandong University, Jinan, Shandong, 250012, China; 2 Department of Joint Surgery, Shandong Provincial Hospital Affiliated to Shandong First Medical University, Jinan, Shandong, 250021, China

**Keywords:** Cartilage, Cytokines, High tibial osteotomy, Osteoarthritis, Platelet-rich plasma.

## Abstract

High tibial osteotomy (HTO) has been widely applied in clinical practice to treat unilateral knee osteoarthritis (OA). In order to improve the effectiveness of surgical treatment, researchers attempted to use a combination of platelet-rich plasma (PRP) and HTO therapy. We summarized the clinical outcomes of engaging HTO and PRP and found promising clinical advantages in improving postoperative pain and function, promoting cartilage repair, and increasing bone cartilage structure in patients. Further studies are needed to provide more convincing evidence of the efficacy of the combined therapy.

## Introduction

Osteoarthritis (OA) is a common degenerative disease marked by cartilage damage and also involves the entire joint, including the articular cartilage, ligaments, synovial membrane, subchondral bone, and surrounding muscles^1^-^4^. In older adults, OA is the primary cause of disability, and it has become more and more common with the progressive aging of the population and the increasing incidence of obesity in China^5^, ^6^. As the most common type of OA, knee OA is mainly characterized by pain, swelling, deformity, and functional impairment of the knee joint. Knee OA is the main source of knee joint pain^6^-^8^. Due to the uncertainty of the etiology and the irreversibility of cartilage damage, the treatment of OA remains challenging. At present, adopting a tiered and personalized treatment plan depends on multiple factors including patient age, gender, lesion location, and severity has become a consensus both domestically and internationally, in order to correct deformities, reduce patient pain, improve joint function, delay disease progression, and improving patient quality of life^7^, ^9^-^11^.

By performing high tibial osteotomy (HTO), the force line of the varus knee joint is restored to normal so as to improve the pain and deformity of the knee joint and slow down the degeneration rate of joint cartilage^12^, ^13^. Due to the fact that HTO does not affect important structures within the joint and preserves the normal movement function of the knee joint as much as possible, it is also known as knee-conserving surgery. There are more and more clinical studies that suggest that the exposed subchondral bone surface can regenerate and cover fibrocartilage under decompression after HTO 14. HTO not only redistributes the load on the knee joint and lessens the pressure on the injured area but also improves the mechanical conditions for cartilage regeneration, which is conducive to the regeneration and repair of cartilage. However, damaged articular cartilage possesses a limited capacity for regeneration and repair. Although the surgical trauma is minimal and there is less intraoperative bleeding, there are still some postoperative complications, including postoperative pain and nonunion of the osteotomy site^15^. In order to promote cartilage regeneration and improve patient prognosis after HTO, researchers attempted to combine intra-articular injection of biologics with traditional HTO, which provides a new possibility to improve the clinical effect of HTO^16^, ^17^.

Autologous platelet-rich plasma (PRP) is the liquid part extracted from one's own autologous peripheral blood, and its platelet concentration is above the baseline 18. PRP has been extensively applied in the treatment of various orthopedic diseases, for example, OA^19^, anterior cruciate ligament reconstruction^20^, ^21^, and tendinopathy^22^. The fundamental scientific principle behind PRP treatment is that injecting PRP at the lesion site can activate tissue repair by releasing many bioactive factors, such as cytokines, growth factors, and adhesive proteins. These bioactive factors and adhesion proteins initiate the revascularization, new connective tissue synthesis, and hemostatic cascade reaction^23^. The migration and proliferation of osteoblasts and chondrocytes can be promoted by the growth factors rich in PRP through activating mesenchymal stem cells (MSCs)^24^, ^25^. These factors promote angiogenesis and bone healing, together with the regeneration and repair of joint cartilage.

The objective of this review is to summarize the application of PRP in HTO, in order to provide more options for improving surgical treatment efficacy and improving patient prognosis.

## Preparation of platelet-rich plasma (PRP)

PRP is prepared from highly concentrated platelet samples attained by centrifuging peripheral blood. After activation through endogenous or exogenous pathways, platelets release biologically active growth factors through degranulation. The released growth factors include platelet-derived growth factor (PDGF), vascular endothelial growth factor (VEGF), insulin-like growth factor (IGF), fibroblast growth factor (FGF), transforming growth factor β(TGF-β), hepatocyte growth factor (HGF) and epidermal growth factor (EGF)(Table 1)^26^, ^27^. PRP exerts various effects, including coagulation, enhanced synthetic metabolism, bone remodeling, angiogenesis, and cell differentiation with the assistance of multiple growth factors 28. The activation of PRP is typically achieved through the utilization of thrombin, calcium chloride, or calcium gluconate 29. PRP can be further classified into four distinct types based on the relative content of platelets, leukocytes, and fibrin: pure platelet-rich fibrin (P-PRF), pure PRP (P-PRP), leukocyte-rich PRP (L-PRP), and leukocyte-rich platelet-rich fibrin (L-PRF) 30. The final PRP product varies due to differences in separation methods, centrifugation speed, and centrifugation time during the production process. First, the collection of whole blood should be undertaken in a test tube that has been treated with an anticoagulant. Citrate, a widely used anticoagulant, disrupts the coagulation cascade by binding to calcium present in the blood. As shown in the graphical abstract, whole blood is divided into three distinct layers after the initial centrifugation. The topmost layer is the plasma layer, which is a pale-yellow transparent liquid, accounting for 55% to 60% of the total volume. The middle layer is the buffy coat, which mainly contains platelets and white blood cells^31^, ^32^. The bottom layer is mainly composed of red blood cells. Centrifuge again after removing the red blood cell layer. After the second centrifugation, the solution can be further separated into two layers. After removing the upper platelet-poor plasma (PPP), the remaining fluid is PRP. The rotational speed and centrifugation time during the preparation of PRP vary in different studies. At present, there is no standardized preparation process for PRP 33.

## High tibial osteotomy (HTO)

The upper tibial osteotomy was initially put forward by Jackson in 1958 as a treatment for unicompartmental knee OA 34. In 1965, Coventry proposed horizontal osteotomy above the tibial tubercle, known as HTO, which became an early standard surgical procedure 35. By transferring the lower limb force line to the neutral position or contralateral compartment, the weight-bearing load on the affected compartment can be reduced, cartilage degeneration can be slowed down, and the demand for total knee arthroplasty can be delayed^36^, ^37^. HTO has been used for the treatment of knee OA for over 50 years.

### Clinical indications

The indications for HTO include isolated lateral compartment OA, isolated medial compartment OA, posterolateral instability OA, adult osteochondritis dissecans, and articular cartilage resurfacing. The HTO procedure is indicated for young, active, non-obese patients with unicompartmental knee OA related to knee malalignment^37^, ^38^. Simultaneously, the following conditions must be met: flexion contracture deformity less than 15°, tibial inversion deformity greater than 5°, medial proximal tibial angle (MPTA) less than 85°, and normal cartilage and meniscus function in the lateral compartment.

### Preoperative evaluation

**Figure 1:**
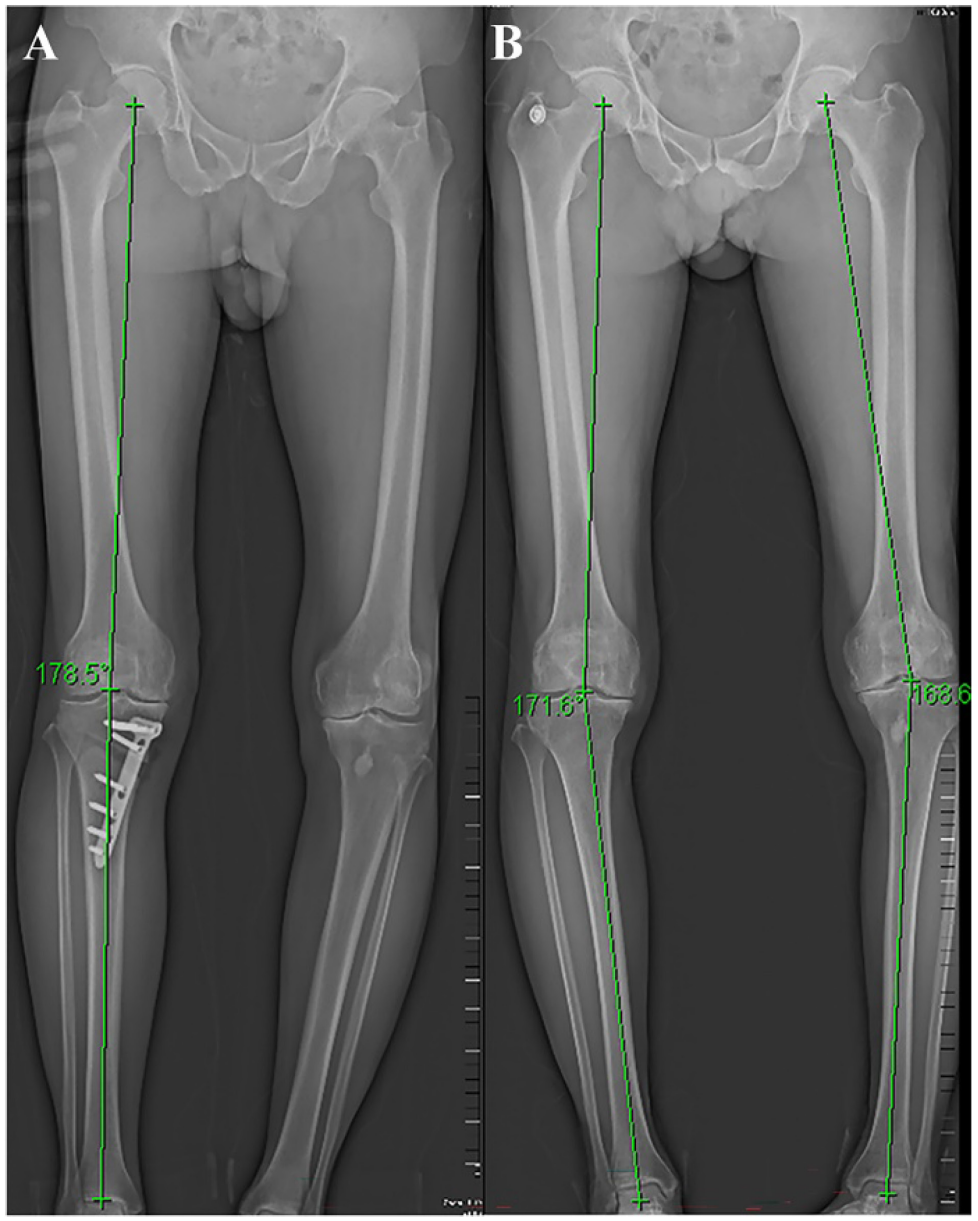
Postoperative (A) and preoperative (B) lower limb X-ray and force line.

Preoperative evaluation includes clinical evaluation and imaging evaluation. Clinical evaluation mainly focuses on the collection of their medical history, physical examination, clinical symptoms, etc. Focus on the precise area of the patient's pain, factors that worsen the pain, and whether there is joint locking or instability. The patella, meniscus, and ligaments should also be evaluated. Patients with the following symptoms are not suitable for surgery: pain that is not consistent with the lesion site, symptomatic patellofemoral degeneration, and severe damage to the meniscus or ligaments. Imaging evaluation was mainly achieved by full-length radiographs of the lower limb, along with front and lateral radiographs of the knee joint (Figure 1). Surgeons use preoperative X-rays to accurately evaluate the lower limb force line (Mikulicz line) and the angle that needs to be corrected 39. CT scanning can better display bone structure, and MRI scanning can better observe the condition of the meniscus, ligaments, and cartilage. Accurate preoperative imaging examination is crucial for evaluating the patient's condition, determining surgical indications, and selecting surgical methods.

### Surgical Technique

**Figure 2:**
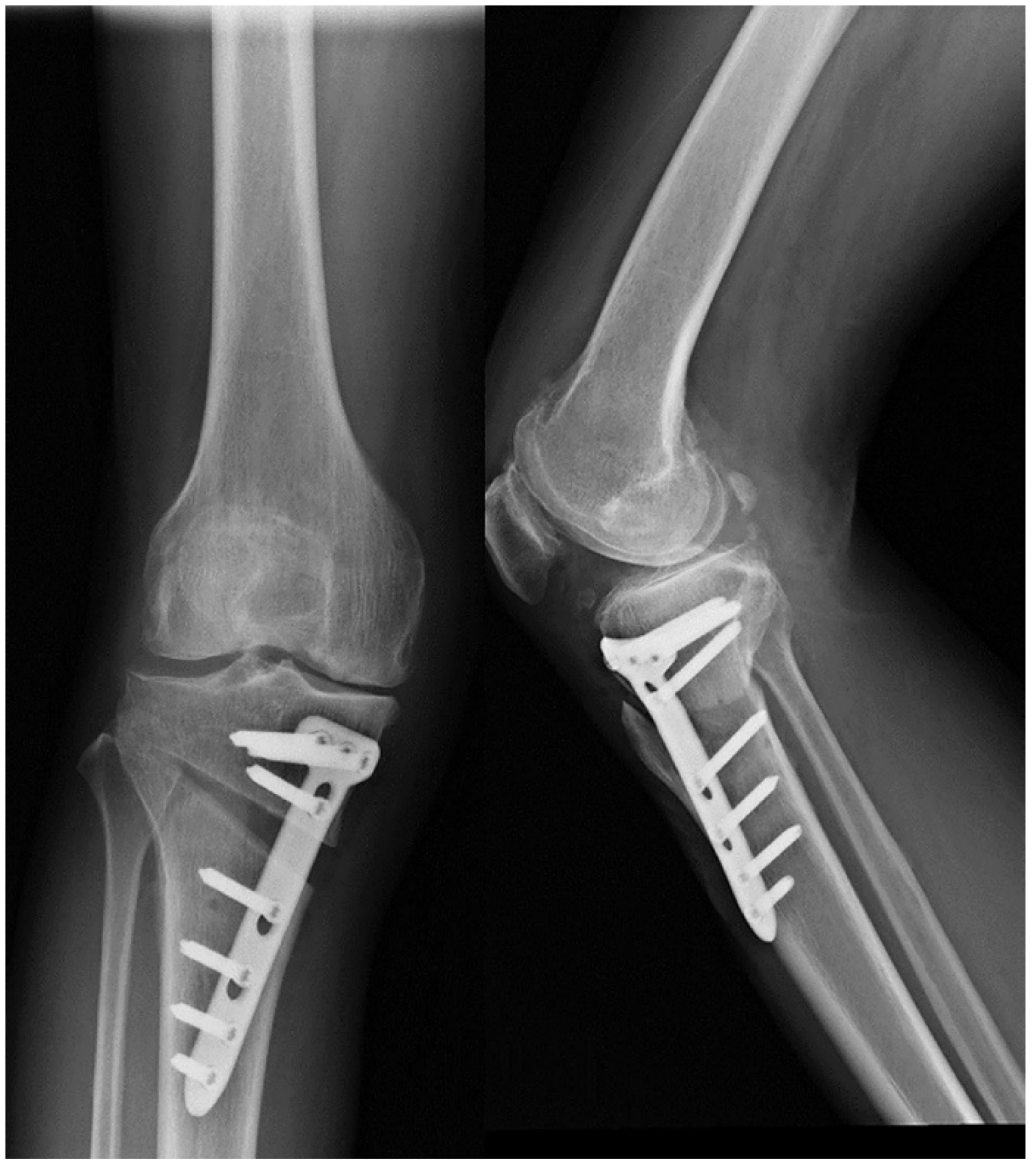
Postoperative X-ray of the knee joint in both anteroposterior and lateral positions after HTO.

The HTO surgical methods include: closing wedge osteotomy, opening wedge osteotomy, dome osteotomy, and “en chevron” osteotomy. The most commonly applied surgical methods are medial opening wedge osteotomy and lateral closing wedge osteotomy. Firstly, a 5-cm longitudinal incision should be made along the leading edge of the pes in the medial upper tibia. The pes was distally retracted to expose the superficial medial collateral ligament, and the patellar ligament was pried open with a blunt pry plate to reveal the tibial tubercle. Using the fibula head as a reference hinge exit point, two 2.5cm Kirschner needles were inserted into the medial tibia at 3cm from the tibial plateau. The two Kirschner needles must be parallel and pointed at the hinge pivot point identified before surgery. The X-ray fluoroscopy was used to confirm accurate positioning and an optimal osteotomy surface. The osteotomy should be performed along the osteotomy line, with approximately 1 cm of the lateral tibial hinge cortex area being preserved. Slowly open the medial osteotomy gap using the “Stacking osteotomes” method 40. Fluoroscopy should be used to ensure whether the lower limb mechanical axis has been correctly corrected (the lower limb force line passes through the Fujisawa point). Insert Tomofix steel plate, drill holes in sequence, and secure with screws. Confirming through fluoroscopy that the position of the steel plate screws is well, and suture the wound layer by layer after rinsing (Figure 2).

### Postoperative treatment and evaluation

Postoperatively, elevate the affected limb, apply ice packs locally to the affected area, and implement multimodal analgesia. Antibacterial drugs are routinely applied to prevent infection, and anticoagulant drugs such as rivaroxaban or low-molecular-weight heparin are used to prevent thrombosis^41^. After the surgical procedure, perform quadriceps functional exercises, ankle pump exercises, and start joint flexion and extension exercises^42^, ^43^. With the aid of a walker, patients were allowed to engage in partial weight-bearing activities^43^. The patients were allowed to gradually increase the load after 6 weeks until it is fully loaded 44. Measure the MPTA and femoral tibial angle (FTA) and evaluate the healing of the osteotomy site through weight-bearing X-rays of bilateral lower limbs. Further guide the functional exercises of the patients based on the healing situation. During postoperative follow-up, assessment scales such as the Visual Analog Score (VAS), Western Ontario and McMaster Universities Osteoarthritis Index (WOMAC), knee injury and osteoarthritis outcome score (KOOS) can be used to evaluate the pain and function of the knee joint^45^.

## HTO combined with PRP therapy

Venous blood was drawn from the elbow vein of the patient in advance for the preparation of PRP. After the surgical operation is completed, the wound is rinsed with normal saline, the prepared PRP or PRP gel is injected into the osteotomy site, and then the wound is sutured layer by layer, with a local pressure dressing^46^, ^47^. After closing the incision, the surgeon injects the prepared PRP into the joint cavity through the joint space adjacent to the patellar ligament. If there is a lot of fluid accumulation in the joint cavity, some of the fluid can be extracted first. Slowly move the knee joint after injection to fully cover the joint surface with PRP and instruct the patient to rest 48. Most studies performed PRP treatment on patients immediately after the operation, while researchers such as Aleksey Prizov chose to perform PRP treatment on patients at 6 weeks after the operation^49^. The treatment cycle and number of treatments of PRP varied from study to study. At present, no standardized treatment protocol exists. However, multiple organizations, including ESSKA, have provided recommendations based on clinical evidence^50^, ^51^.

## The effects of HTO combined with PRP

### PRP can improve pain and function after HTO

It has been demonstrated by preceding studies that PRP can alleviate pain and symptoms in patients with OA^52^, ^53^. IGF-1 or PDGFB, which is rich in PRP, can impede the activation of NF-κB by inhibiting κB kinase alpha (IKKα). Subsequently, it inhibits the transcription of downstream signaling molecules of NF-κB, which are involved in the process of inflammation and cell apoptosis, and reverses the anti-anabolic effect of IL-1β 54. PRP can alleviate pain through the reduction of inflammation and angiogenesis in the synovium, which is the location of pain receptors 55. Peter Everts et al. noticed that the analgesic effect of PRP may be associated with the pain regulatory effect of its derivative 5-HT^23^. However, the specific molecular mechanism of PRP-mediated pain regulation still needs to be clarified by further clinical studies. PRP can promote the synthesis of HGF and hyaluronic acid by synovial cells. HGF has been demonstrated to have the capacity to limit the inflammatory response within the synovium^56^, ^57^. The improvement of postoperative clinical results of patients with PRP is related to its anti-inflammatory effect, antibacterial effect, and pain relief. The results of a meta-analysis show that PRP can alleviate pain in patients with various musculoskeletal disorders^58^. Except for orthopedic diseases, PRP has also demonstrated therapeutic advantages in various systemic diseases^59^, ^60^.

Multiple studies investigated whether using PRP treatment after HTO surgery could improve pain and functional outcomes. Dong and his colleagues 48 used the VAS and WOMAC scores to investigate the clinical outcome of a treatment combining HTO with PRP intervention in the therapy of severe knee OA. They reported that compared with the control group, the HTO + PRP group demonstrated a marked decrease in VAS scores and WOMAC scores in the results of the final follow-up. The VAS scores and WOMAC scores of patients treated with HTO + PRP are superior to patients who received HTO treatment alone. Combined treatment of HTO and PRP therapy may provide a feasible treatment option to address knee pain and slow down the progression of knee OA. Zhang et al. 61 conducted a randomized controlled trial in which they randomly assigned 80 patients to two groups. Among them, 39 patients received combined treatment with HTO and PRP, while the remaining 41 patients received only HTO. The WOMAC score, the VAS score, and the Lysholm score were implemented to prospectively evaluate patients in both groups before surgery, and post-operative follow-ups were conducted at 1-, 6-, 12-, and 24-months post-surgery. The follow-up data shows that the joint pain and function of the treatment group patients were significantly improved, especially at six months, the WOMAC score (HTO+PRP: 75.6 ± 15.4; HTO alone: 90.3 ± to 11.9; P<0.001) and Lysholm score (HTO+PRP: 83.1 ± 14.7; HTO alone: 72.5 ± 10.6 points; P<0.001). However, the advantage of the HTO+PRP group in comparison to the HTO alone group with respect to pain and functional scores disappeared at the final follow-up. In addition, multiple randomized controlled trials^47^, ^62^-^64^ have used various knee pain function scores (such as VAS, WOAMC, and KOOS) to investigate the clinical efficacy of HTO alone and HTO along with PRP treatment. In comparison with the control group (HTO alone), HTO along with PRP treatment achieved better results, and a statistically significant difference was noted between the two groups. Yong Gon Koh et al.^16^ investigated the clinical outcomes of the utilization of PRP and PRP in conjunction with MSCs for treating HTO. The KOOS score, Lysholm score, and VAS score were used to evaluate postoperative pain and function in patients. Moreover, it was also determined that the combination of HTO and PRP-MSC exhibited a more pronounced improvement trend in the VAS score and Koos score scale. Significant differences were observed in the pain and symptom scales between the HTO + PRP and HTO + PRP-MSC groups at the final follow-up. A significant difference was noted in the average VAS scores between the two groups (P < 0.01), but no significant difference was observed in the Lysholm scores among the two groups (P = 0.36). Aleksey Prizov et al.^65^ also used KOOS, KSS, and VAS scores to assess the therapeutic efficacy of PRP or stromal vascular fraction (SVF) treatment in HTO patients after surgery. When compared to preoperative status, the two groups showed statistically significant improvement in knee joint function, and PRP treatment achieved better clinical outcomes compared to SVF. The VAS scores and IKDC scores of HTO combined with PRP treatment were better than sodium hyaluronate treatment at 1 and 2 years after surgery^46^.

### PRP promotes cartilage repair

The promotion of cartilage growth by PRP is closely linked to the growth factors it contains, for instance, FGF, PDGF, TGF-β, etc. 54 PRP can promote the synthesis of proteoglycans, inhibit the breakdown metabolism of proteoglycans, and enhance the deposition of extracellular matrix^66^, ^67^. PRP can promote recruitment and the migration of MSCs to the repair site. PRP has the capacity to induce the differentiation of bone marrow mesenchymal stem cells (BMSCs) into cartilage cells, enhance chondrocyte vitality, and promote chondrocyte proliferation 55. PRP could increase the expression of the proteoglycan gene, increase type II collagen, and downregulate the expression of the type I collagen gene. In OA and cartilage defect animal models, the PRP treatment group achieved better cartilage regeneration, overall appearance, as well as histological and immunohistochemical characteristics 29.

Zhang et al. 61 evaluated postoperative cartilage regeneration by preoperative arthroscopy and postoperative MRI measurement of cartilage thickness. In addition, the minimum width of the medial knee joint (MJSW) was calculated by taking full-length EOS images of the patient's lower limbs. The patients were randomly distributed across two groups: HTO combined with PRP treatment and HTO alone. The data shows that the MJSW values of the HTO+PRP group improved by 0.8, 0.8, 0.4, and 0.2mm, respectively, compared to the HTO alone group at 1, 6, 12, and 24 months postoperatively. This indicates that compared to HTO alone treatment, the combined use of HTO and PRP significantly improved MJSW. Dong et al. 48 performed an arthroscopic examination on patients before the operation and after the removal of steel plates (12 months postoperatively), evaluated the status of articular cartilage using International Cartilage Repair Society (ICRS) grading, and measured the thickness of articular cartilage using MRI during postoperative follow-up. At the conclusion of the follow-up, there were significant differences in the thickness of medial femoral cartilage and ICRS grading between the HTO+PRP group and the control group (HTO+normal saline). There were also significant differences between the HTO+PRP and HTO+hyaluronic acid subgroups. When using PRP treatment, cartilage damage was significantly reduced, and repair of femoral and tibial joint cartilage was observed in all patients. In another randomized controlled trial^46^, PRP showed greater advantages in cartilage regeneration compared to sodium hyaluronate. Yong Gon Koh et al.^16^ used Kanamiya grading to evaluate the healing of articular cartilage. The combined treatment of PRP and MSC after HTO achieved better cartilage repair results compared to using PRP alone. Aleksey Prizov et al.^65^ evaluated cartilage injury using the Outerbridge score under arthroscopy and found that SVF treatment after HTO showed more significant cartilage regeneration compared to PRP treatment. In another of their studies^49^, histological materials containing bone and cartilage were extracted under arthroscopy for bone biopsy. The final experimental results show that all specimens showed transparent fibrous tissue or cartilage at the end. Furthermore, the height of the subchondral bone and cartilage thickness demonstrated favorable outcomes following treatment.

### PRP and postoperative improvement of anatomical axis

Two studies used the FTA and the MPTA to evaluate the improvement of the lower limb mechanical axis after HTO combined with PRP. Their results showed that significant improvements were observed in both MPTA and FTA compared to preoperatively. However, since no blank control group was set, the improvement efficacy of PRP as a standalone treatment modality remains to be demonstrated. Zhang et al. 61 evaluated the improvement of FTA, MPTA, and weight-bearing line (WBL) by taking full-length EOS images of the patient's lower limbs in a randomized controlled trial. In spite of the fact that there were significant improvements in the FTA, MPTA, and WBL in both groups in comparison to preoperative data. Nevertheless, the difference between the two groups (HTO alone and HTO+PRP) is not statistically significant. Another randomized controlled trial in which HTO was performed in the control group and HTO in conjunction with PRP was used in the experimental group, and also found similar results. Yong-Gon Koh et al. also used FTA and WBL to evaluate the improvement of the lower limb force line after HTO treated with PRP alone versus HTO treated with PRP combined with MSCs. At the conclusion of the follow-up, the combination of HTO and PRP demonstrated significant improvement in FTA and WBL in patients compared with preoperative treatment. A comparison of the postoperative improvement in FTA and WBL between the PRP+MSC combination therapy and PRP alone revealed no significant differences.

Based on existing evidence, no advantage of PRP in promoting the improvement of the lower limb line of force after HTO surgery has been found. Although Zhang et al. discovered that PRP has the capacity to enhance the MJSW after HTO surgery, the present studies have failed to find evidence to suggest a relationship between MJSW and the improvement of the anatomical axis.

### HTO combined with PRP can increase and strengthen cartilage and bone architecture

In another study by Aleksey Prizov^49^, researchers extracted histological materials containing bone and cartilage under arthroscopy for bone biopsy. Mainly evaluated trabecular bone volume (Tr.V), bone volume (BV), average intratrabecular space (Tr.Sp), subchondral bone volume (Cr.V), articular cartilage thickness (Ch.Wi), mean number of trabeculae (Tr. N), etc. At the final follow-up in the PRP treatment group, significant increases in Tr.V and mean number of Tr.N were observed in both the medial tibial condyle and medial femoral condyle. In addition to changes in bone units, significant changes in cartilage thickness and subchondral bone height were also observed after treatment. This article studied the efficacy of PRP treatment on bone and cartilage morphology and morphometric parameters after long-term HTO. It has been proven that HTO surgery combined with PRP treatment can significantly enhance the cartilage and bone structure of knee joints in patients with knee arthritis.

### PRP and bone healing

In the past few years, the application of PRP with the intention of accelerating the induction of fracture healing has received widespread attention. Due to its rich content of growth factors such as PDGE, VEGF, and TGF-β, PRP has been proven to promote bone healing *in vitro* experiments^68^-^70^. The growth factors secreted by PRP have the ability to promote angiogenesis and activate the proliferation and chemotaxis of MSCs, osteoblasts, and chondrocytes^25^, ^71^, ^72^. One of the components of PRP, bone morphogenetic protein-2 (BMP2), has been demonstrated to be capable of inducing osteogenic differentiation of MSCs, thereby promoting the formation of new bone and revascularization^73^. PRP is a powerful angiogenic agent that coordinates angiogenesis by promoting endothelial cell migration and proliferation, recruiting vascular smooth muscle cells, etc. 74 The VEGF rich in PRP can promote bone remodeling and bone matrix mineralization by enhancing osteoclast activity and chemotaxis and recruiting osteoblasts^75^, ^76^. There is evidence to support the application of PRP in promoting the healing of mandibular fractures and the integration of allogeneic bone^69^, ^77^. The results of a meta-analysis show that PRP can enhance the fracture healing rate and reduce delayed fracture healing 78.

Dallari D et al. 79 completed a prospective randomized controlled trial. During tibial wedge osteotomy, lyophilized bone chips with platelet gel and lyophilized bone chips with platelet gel combined with BMSCs were used to fill the bone defect intensively. Lyophilized bone chips were the only treatment given to the control group. Platelet gel is formed by mixing 4ml thrombin with 16ml PRP from patients. Thrombin is a platelet activator that can effectively induce PRP to initiate the release of growth factors^72^, ^80^. At one and a half months, three months, six months, and twelve months after surgery, clinical, radiological, and histopathological evaluations were conducted on patients through X-ray imaging and needle aspiration biopsy of the implantation site. Six weeks after surgery, the results indicated a substantial enhancement in the bone integration process in both experimental groups in comparison with the control group. The emergence of new osteoblasts and extensive bone formation were observed at the implantation site. In comparison with the control group, the two experimental groups had more active osteogenesis. Compared with the lyophilized bone chips + platelet gel group, the osteogenesis of the lyophilized bone chips + platelet gel + BMSCs group was more active. Similar to the above research results, the composite graft scaffold doped with PRP can shorten the healing time of fractures and demonstrate preferable biological advantages^81^, ^82^. Liu et al. measured the average bone density in the osteotomy area by taking CT scans of the patient's knee joint and indirectly determined the growth of new bone in the osteotomy area. At three months after surgery, a noteworthy increase in bone density (CT value) was noted in the osteotomy area of the PRP group in comparison with the control group. There was a significant statistical difference between the two groups 47.

In the retrospective study by Steven A. Giuseff et al., out of 89 cases of osteotomy, 5 cases experienced nonunion, with 2 cases occurring in the allogeneic bone +PRP group 83. Their research results show that allograft combined with demineralized bone matrix and/or PRP was related to nonunion. As this is a retrospective study, there was no control group set up in the experiment, and the sample size was small, so there is a high risk of accidental bias. The research results need stronger evidence to support them. Bone transplantation can promote the formation of new bone through osteogenic stimulation, bone conduction stimulation, and bone induction stimulation. Autologous iliac bone graft is the most commonly used bone graft. Caio Oliveira D'Elia et al. explored whether PRP and bone marrow aspiration mixture could be used in place of autologous iliac bone grafts in HTO 84. The experimental group used PRP and bone marrow aspiration mixture, while the control group applied autologous iliac bone grafts. Unfortunately, compared to autologous iliac bone grafts, the PRP+ bone marrow aspiration mixture did not show any advantages. Although some studies have indicated the possible function of PRP in promoting HTO bone healing, the specific mechanism still needs further exploration. Currently, most studies lack randomized controlled clinical trials (I-II level evidence), and there is a need for larger sample sizes and more standardized scientific research on the application of PRP to demonstrate its true clinical effectiveness in promoting bone healing.

### PRP and cytokines

Aleksey Prizov et al. first investigated the impact of PRP injection after HTO surgery on the release of cytokines in synovial fluid of people suffering from knee OA and analyzed the cytokine profile of synovial cells related to long-term clinical outcome evaluation of patients^65^. Researchers collected synovial fluid from patients before and after PRP injection therapy and analyzed the levels of cytokines in it. After PRP treatment, the levels of IL-6 and interferon-gamma inducible protein-10 (IP-10) in the patient's synovial fluid significantly decreased, while the expression of sCD40L and PDGF-AB/BB was upregulated. IL-6 is a pro-inflammatory cytokine that can trigger osteoclast differentiation and bone resorption, playing a role in inflammation and degenerative joint diseases 85. PDGF-AB/BB is a double-stranded peptide belonging to the growth factor family that can stimulate bone DNA and protein synthesis. It may act as a regulator of bone growth, exerting effects throughout the body or locally. PDGF-AB/BB is released as a systemic growth factor in the process of platelet aggregation. It has a key role in the initial phase of fracture healing 86. sCD40L plays a crucial role in immune response and inflammatory processes, and its abnormal expression is closely linked with the progression of a range of illnesses. It is considered to belong to the medium cluster related to growth factors 87. However, there is no research yet to prove its specific role in the initiation and progression of knee joint diseases. In Yan et al.'s retrospective study 88, patients treated with HTO combined with PRP had significantly lower postoperative levels of TNF-α and IL-6 in comparison with preoperative levels, while PDGF, EGF, and VEGF levels were significantly higher than preoperative levels.

## Prospects and Challenges

Although most experimental results indicate that PRP treatment can improve knee joint pain and function in patients after HTO surgery, it can achieve better clinical treatment outcomes. However, there are no standardized treatment protocols to prove a stable therapeutic effect yet. The main limitations are small sample size, short follow-up time, and different treatment plans. The optimal injection timing and frequency of PRP treatment need to be further explored. Although PRP has shown the potential to promote cartilage repair, the long-term evidence from MRI or arthroscopy is still insufficient. Some studies lack blank controls, thus failing to reflect the true effectiveness of PRP treatment. So, it's important to distinguish between the established role of HTO and the more therapeutic role of PRP in this context. A large number of standardized randomized controlled trials are needed to verify the efficacy of HTO combined with PRP treatment. Although most studies have not reported the adverse reactions of PRP treatment, some possible adverse reactions caused by PRP, such as allergic reactions and joint swelling, still need to be paid attention to in future clinical trials^51^. With the development of regenerative medicine, stem cell therapy, tissue engineering, gene therapy, etc., have gradually been applied in clinical practice^89^, ^90^. In the future, the synergistic therapy of PRP combined with stem cells and tissue engineering may bring new options to patients.

## Conclusion

PRP has been extensively applied in diverse fields, bringing new options for the treatment of various diseases. With the continuous deepening of research, the role of PRP has been further explored, and its applications have become more extensive. The combination of PRP treatment for arthritis patients after HTO surgery has opened up a new field of PRP treatment for orthopedic diseases and provided a new option for improving postoperative clinical outcomes. In the current studies, PRP demonstrated satisfactory results in improving pain and function after HTO in patients, promoting cartilage repair and bone healing, improving lower limb lines of force, and enhancing bone structure. The combination of PRP and other biological agents may provide better therapeutic effects. However, the existing number of related studies is relatively small, and further clinical trials are required to verify its effectiveness. The long-term clinical outcomes and adverse reactions of HTO combined with PRP treatment require more large-scale trials and long-term follow-up.

## Figures and Tables

**Table 1:** Growth factors and their functions in platelet-rich plasma (PRP).

Growth Factor	Function in PRP
PDGF	Increases angiogenesis. Promotes the proliferation and differentiation of osteoblasts, fibroblasts, and macrophages. Promotes collagen and matrix synthesis.
TGF-β	Promotes angiogenesis. Promotes collagen and extracellular matrix synthesis. Regulates other growth factors. Stimulates the proliferation and differentiation of chondrocytes and the formation of bone matrix.
VEGF	Increases vascular permeability and promotes angiogenesis. Promotes the migration and proliferation of vascular endothelial cells. Promotes bone remodeling and bone matrix mineralization.
FGF	Promotes angiogenesis. Promotes the proliferation and differentiation of chondrocytes. Fibroblast migration.
EGF	Promote the proliferation and migration of epithelial cells. Stimulate angiogenesis. Promote wound healing.
IGF	Promote chondrocyte proliferation, differentiation, and bone matrix synthesis. Fibroblast chemotaxis. Promote extracellular matrix synthesis.
HGF	Inhibits the NF-κB pathway. Stimulates the growth of endothelial cells. Promotes angiogenesis and mitosis.

## Abbreviations

HTO: High tibial osteotomy
PRP: platelet-rich plasma
OA: osteoarthritis
PDGF: platelet-derived growth factor
VEGF: vascular endothelial growth factor
IGF: insulin-like growth factor
FGF: fibroblast growth factor
TGF-β: transforming growth factor β
HGF: hepatocyte growth factor
EGF: epidermal growth factor
P-PRF: pure platelet-rich fibrin
P-PRP: pure PRP
L-PRP: leukocyte-rich PRP
L-PRF: leukocyte-rich platelet-rich fibrin
PPP: platelet-poor plasma
MPTA: medial proximal tibial angle
IKKα: κB kinase alpha
VAS: Visual Analog Score
WOMAC: Western Ontario and McMaster Universities
MSCs: mesenchymal stem cells
BMSCs: bone marrow mesenchymal stem cells
MISW: minimum width of the medial knee joint
FTA: femoral tibial angle
ICRS: International Cartilage Repair Society
SVF: stromal vascular fraction
WBL: weight-bearing line
Tr.V: trabecular bone volume
BV: bone volume
Tr.Sp: average intratrabecular space
Cr.V: subchondral bone volume
Ch.Wi: articular cartilage thickness
Tr.N: mean number of trabeculae
BMP2: bone morphogenetic protein-2
IP-10: interferon-gamma inducible protein-10
